# Evaluation of antinociceptive, *in-vivo & in-vitro* anti-inflammatory activity of ethanolic extract of *Curcuma zedoaria* rhizome

**DOI:** 10.1186/1472-6882-14-346

**Published:** 2014-09-22

**Authors:** HM Arif Ullah, Sayera Zaman, Fatematuj Juhara, Lucky Akter, Syed Mohammed Tareq, Emranul Haque Masum, Rajib Bhattacharjee

**Affiliations:** College of Pharmacy, Gyeongsang National University, 501 Jinju-daero, Jinju, Gyeongnam, 660-751 Republic of Korea; Department of Pharmaceutical Sciences, School of Life Sciences, North South University, Bashundhara, Dhaka, 1229 Bangladesh; Department of Pharmacy, Faculty of Science and Engineering, Southern University Bangladesh, Chittagong, Bangladesh

**Keywords:** *Curcuma zedoaria*, Antinociceptive activity, *In-vivo & in-vitro* anti-inflammatory activity

## Abstract

**Background:**

The present study was aimed to investigate the antinociceptive and anti-inflammatory activity of the *Curcuma zedoaria* (family Zingiberaceae) ethanolic rhizome extract in laboratory using both *in vitro and in vivo* methods so as to justify its traditional use in the above mentioned pathological conditions.

**Methods:**

Phytochemical screening was done to find the presence of various secondary metabolites of the plant. *In vivo* antinociceptive activity was performed employing the hot plate method, acidic acid induced writhing test and formalin induced writhing test on Swiss albino mice at doses of 250 and 500 mg/kg body weight. Anti-inflammatory activity test was done on Long Evans rats at two different doses (250 and 500 mg/kg body weight) by using carrageenan induced paw edema test. Finally *in vitro* anti-inflammatory test by protein-denaturation method was followed. Data were analyzed by one-way analysis of variance (ANOVA) and Dunnett’s *t*-test was used as the test of significance. P value <0.05 was considered as the minimum level of significance.

**Results:**

Phytochemical screening revealed presence of tannins, saponins, flavonoids, gums & carbohydrates, steroids, alkaloids, reducing sugars and terpenoids in the extract. In the hot plate method, the extract increased the reaction time of heat sensation significantly to 61.99% and 78.22% at the doses of 250 and 500 mg/kg BW respectively. In acetic acid induced writhing test, the percent inhibition of writhing response by the extract was 48.28% and 54.02% at 250 and 500 mg/kg doses respectively (p < 0.001). The extract also significantly inhibited the licking response in both the early phase (64.49%, p < 0.01) and the late phase (62.37%, p < 0.01) in formalin induced writhing test. The extract significantly (p < 0.05, p < 0.01 and p < 0.001) inhibited carrageenan induced inflammatory response in rats in a dose related manner. In in-vitro anti-inflammatory test, the extract significantly inhibited protein denaturation of 77.15, 64.43, 53.04, 36.78 and 23.70% for doses of 500, 400, 300, 200 and 100 μg/mL respectively.

**Conclusions:**

The results obtained from the tests indicate that the plant might have one or more secondary metabolite(s) having central and peripheral analgesic and anti-inflammatory activity.

## Background

The majority population of the developing world relies on traditional herbal medicine as the primary source of treatment for illnesses [1]. The issue of compliance with herbal medicines varies according to local beliefs and socio-cultural status, and is less reliant on the efficacy of the traditional medicine.

About 25% of the drugs prescribed worldwide come from plants, 121 such active compounds being in current use of the 252 drugs considered as basic and essential by the World Health Organization (WHO), 11% are exclusively of plant origin and a significant number are synthetic drugs obtained from natural precursors.

Modern social context and economic view of health services, the needs of the pharmaceutical market and the recognition that research on medicinal plants used in folk medicine represents a suitable approach for the development of new drugs [2, 3] have led to an increase in the number of publications in this field, and private and governmental institutions are now financially supporting research programs worldwide.

It is estimated that, in 1997, the world market for over the-counter phytomedicinal products was US$ 10 billion, with an annual growth of 6.5% [4]. In 2003, growth was well over expectation with sales exceeding USD 65 billion, with USD 9 billion in Europe alone. The WHO considers phytotherapy in its health programs and suggests basic procedures for the validation of drugs from plant origin in developing countries [5]. China and India have a well-established herbal medicines industry and Latin American countries have been investing in research programs in medicinal plants and the standardization and regulation of phytomedicinal products.

As mentioned earlier, interest in herbal medicine as a path to drug development increased greatly in the early 1980s [6]. This could be due to the inefficiency of conventional medicine (e.g. cytotoxicity, side effects and ineffectiveness of synthetic drugs), abusive and incorrect use of synthetic drugs and most importantly, the high cost involved in conventional medicine and the fact that a large percentage of the world’s population does not have access to conventional pharmacological treatment. With the limitations of synthetic chemistry, there also arises the need to find new medicines to combat the emergence of multi-resistant pathogens [6], as well as to manage a whole range of chronic and difficult-to-treat diseases such as cancer, diabetes and AIDS. Natural products offer unmatched structural variety and their usefulness can be extended by probing biological pathways [7].

Inflammation is a complex biological response of vascular tissues to harmful stimuli. It is also a protective attempt by the organism to remove the injurious stimuli and initiate the healing process [8]. At the onset of an inflammation, the cells undergo activation and release inflammatory mediators. These mediators include histamine, serotonin, slow reacting substances of anaphylaxis (SRS-A), prostaglandins and some plasma enzyme systems such as the complement system, the clotting system, the fibrinolytic system and the kinin system [9]. These mediator molecules work collectively to cause increased vasodilatation and permeability of blood vessels. Thus, leading to increased blood flow, exudation of plasma proteins and fluids, and migration of leukocytes, mainly neutrophils, outside the blood vessels into the injured tissues. Inflammation can be classified as either acute or chronic inflammation [8]. Acute inflammation is the initial response of the body to injurious stimuli and is achieved by increased movement of plasma and leukocytes from the blood into the injured tissues. The process of acute inflammation is initiated by cells already present in the tissues. This is characterized by marked vascular changes, including vasodilatation and increased capillary permeability which are induced by the actions of the various inflammatory mediators. Chronic inflammation is a prolonged inflammatory response that leads to a progressive shift in the type of cells present at the site of inflammation and is characterized by simultaneous destruction and healing of the tissues from the inflammatory process.

Zedoary (*Curcuma zedoaria*) is the name for a perennial herb and member of the genus Curcuma Linn. Family: Zingiberaceae. It is also known as White Turmeric. The plant is native to India and Indonesia. It was introduced to Europe by Arabs around the sixth century, but its use as a spice in the West today is extremely rare, having been replaced by ginger.

Zedoary, also known as white turmeric, is a rhizome with a thin brown skin and a bright orange, hard interior. Its smell is similar to that of turmeric and mango. The perennial herb has a warm-spicy, woody and camphoraceous cineolic odor and bears yellow shiny flowers, with red and green bracts. The ovate leaves possess purple-colored spots and are 1 to 2 feet long, narrowing at the base.

Traditionally the plant is used to for a variety of disease conditions. The paste prepared from the plant is used to treat inflammation, pain, wounds and skin ailments. Power prepared from the dried plant is used to treat menstrual irregularities. The bitter tincture of zedoary rhizome is used for the treatment of recurring diseases like malaria fever. It is also used to treat ulcers.

Previous works investigating analgesic property [10] and anti-inflammatory potential [11] carried out using extracts from different parts of the plant on laboratory animals. Therefore the present study was undertaken employing both *in vitro* and *in vivo* methods to explore possible antinociceptive and anti-inflammatory potential of the ethanolic rhizome extract of *Curcuma zedoaria* so as to establish a plausible mechanism of action of extract and to justify the traditional uses of this plant.

## Methods

### Collection and proper identification of the plant sample

The plant was *Curcuma zedoaria.* It was collected from Savar, Bangladesh during the month of February. The whole plant was collected and sun dried. The plant was identified by the experts of Bangladesh National Herbarium (BNH), Mirpur, Dhaka and was given an accession number which was 38765. The specimen was preserved in BNH.

### Preparation of powdered plant material

The collected plant was washed with water, separated from undesirable materials. They were put under sunshade to be partially dried. Then they were heated through oven to be fully dried at below 40°C for two days. The fully dried leaves were then grinded to make them powder by the help of a suitable grinder. The powder obtained was extracted via the method of cold extraction using ethanol and then kept for a period of 5 days accompanying occasional shaking and stirring. The whole mixture then underwent a coarse filtration through a piece of clean, white cotton material followed by a second filtration through Whatman no. 1 filter paper. The filtrate (ethanol extract) obtained was evaporated by rotary evaporator (Bibby RE-200, Sterilin Ltd., UK) at 5 to 6 rpm and at 68°C temperature. It rendered a gummy concentrate of dark greenish-black color that was designated as crude ethanolic extract. The extract was finally dried by a freeze drier and preserved.

### Animals

Young Swiss-Albino mice aged about 4–5 weeks with average weight of 25–35 gm and adult Long Evans Rats of either sex having average weight of 100–130 gm were used for the experiment and maintained in the animal house of the Department of Pharmacy, North South University for acclimation. The animals were originally obtained from International Centre for Diarrheal Disease Research, Bangladesh (*ICDDR*, *B*). They were housed in standard cages under standard environmental conditions of room temperature at 24 ± 1°C and 55-65% relative humidity with 12 hour dark light cycle and provided with standard food for rodents and water *ad libitum*. The experimental protocol was approved by the Ethics committee of the North South University and was performed according to the Guidelines for Animal experimentation established by the International Centre for Diarrhoeal Disease Research, Bangladesh (*ICDDR*, *B*).

### Method for phytochemical analysis

The freshly prepared extract of *C. zedoaria* was qualitatively tested for the presence of chemical constituents. Qualitative phytochemical tests for the identification of alkaloids, flavonoids, steroids, gum and carbohydrates, saponins, reducing sugar, tannins and terpenoids were carried out for the extract by the method described previously [12].

### Method for the evaluation of analgesic effect

#### Hot plate test

The hot-plate test (Hot/Cold Plate Model-35100-001, UGO Basile, Italy) was employed for measurement of analgesic activity as previously described by Lanhers et al*.* and modified by Ojewole [13, 14]. The temperature was regulated at 55° ± 1°C. Mice of either sex were divided into four groups consisting of six animals in each group. The mice of each group were placed in the beaker (on the hot plate) in order to obtain its response to electrical heat induced pain stimulus. Licking of the paws or jumping out of the beaker was taken as an indicator of the animal’s response to heat-induced pain stimulus. The time for each mouse to lick its paws or jump out of the beaker was taken as reaction time (in second). Before treatment, the reaction time was taken once. The mean of this determination constituted initial reaction time before treatment of each group of mice. Each of the test mice was thereafter treated with either distilled water (DW), Diclofenac sodium (10 mg/kg BW) or ethanol extract of *C. zedoaria* at the doses of 250 and 500 mg/kg BW orally. Thirty minutes after treatment, the reaction times of each group of mice were again evaluated five times individually on one hour interval. Percent analgesic score was calculated as,


Where, *T*_*b*_ = Reaction time (in second) before drug administration; *T*_*a*_ = Reaction time (in second) after drug administration.

#### Acetic acid-induced writhing method

The analgesic activity of the sample was evaluated using acetic acid induced writhing method in mice following the method of Koster et al. with slight modification [15–17]. In this method, acetic acid is administered intraperitoneally to the experimental animals to create pain sensation. The animals were divided into four groups with six mice in each group. Group I animals received distilled water, Group II received Diclofenac sodium at 10 mg/kg while animals of Group III and Group IV were treated with 250 and 500 mg/kg of the ethanol extract of *C. zedoaria* after an overnight fast. Test samples and vehicle were administered orally 30 minutes prior to intraperitoneal administration of 0.7% v/v acetic acid solution. Animals were kept individually under glass jar for observation. Each mouse of all groups was observed individually for counting the number of writhing they made in 10 minutes commencing just 5 minutes after the intraperitoneal administration of acetic acid solution. Full writhing was not always accomplished by the animal, because sometimes the animals started to give writhing but they did not complete it. This incomplete writhing was considered as half-writhing. Accordingly, two half-writhings were taken as one full writhing. The number of writhes in each treated group was compared to that of a control group where Diclofenac sodium was used as a reference standard (positive control). The percentage inhibition of writhing was calculated as follows:


*VT* = number of writhing motions in extract-treated mice.

*VC* = number of writhing motions in the control group of mice.

#### Formalin induced writhing test

The method used was similar to that described previously [18, 19]. The mice were divided into four groups each containing 6 mice and were administered with either distilled water (1 ml/kg, *i.p.*), ethanolic extract of *C. zedoaria* (250 and 500 mg/kg, *i.p*) or Diclofenac sodium (10 mg/kg, *s.c*). Thirty minutes after this treatment; 50 μL of a freshly prepared 0.6% solution of formalin was injected subcutaneously under the plantar surface of the left hind paw of each mice. The mice were placed individually in an observation chamber and monitored for one hour. The time (in second) spent in licking and biting responses of the injected paw was taken as an indicator of pain response. Anti-nociceptive effect was determined in two phases. The early phase (phase 1) was recorded during the first 5 minutes, while the late phase (phase 2) was recorded during the last 20–30 minutes after formalin injection.

### Method for the evaluation of anti-inflammatory effect

#### Evaluation of in vivo anti-inflammatory effect

The anti-inflammatory activity of the ethanol extract was investigated on carrageenan induced inflammation in rat paw following an established method [20]. Rats were randomly divided into four groups, each consisting of six animals, of which group I was kept as control giving only distilled water. Group II was standard which received Diclofenac sodium (10 mg/kg) as the reference standard for comparison while Group III and Group IV were given the test material at a dose of 250 and 500 mg/kg body weight respectively. Half an hour after the oral administration of the test materials, 1% carrageenan was injected to the right hind paw of each animal. The volume of paw edema was measured at 0, 1, 2, 3, 6 and 9 hours using Plethysmometer (Model 7141, UGO Basile, Italy) after administration of carrageenan. The left hind paw served as a reference non-inflamed paw for comparison.

The average percent increase in paw volume with time was calculated and compared against the control group. Percent inhibition was calculated using the formula-


Where, *V*_*c*_ and *V*_*t*_ represent average paw volume of control and treated animal respectively.

#### Evaluation of in vitro anti-inflammatory activity

The reaction mixture (5 mL) consisted of 0.2 mL of egg albumin (from fresh hen’s egg), 2.8 mL of phosphate buffered saline (PBS, pH 6.4) and 2 mL of varying concentrations of extract so that final concentrations become 100, 200, 300, 400, 500 μg/mL. Similar volume of double-distilled water served as control. Then the mixtures were incubated at (37°C ± 2) in a BOD incubator (Lab line Technologies) for 15 min and then heated at 70°C for 5 min. After cooling, their absorbance was measured at 660 nm (SHIMADZU, UV 1800) by using vehicle as blank. Acetyl salicylic Acid at the final concentration of (100, 200, 300, 400, 500μg/mL) was used as reference drug and treated similarly for determination of absorbance.

The percentage inhibition of protein denaturation was calculated by using the following formula:


### Statistical analysis

The data are expressed as the mean ± SEM analyzed by one-way analysis of variance (ANOVA) and Dunnett’s *t*-test was used as the test of significance. P value <0.05 was considered as the minimum level of significance. All statistical tests were carried out using SPSS statistical software.

## Results

### Phytochemical analysis

Phytochemical screening of ethanol extract of *C. zedoaria* revealed the presence of various bioactive components of which tannins, saponins, gums & carbohydrates, reducing sugar, alkaloid, steroids and terpenoids were the most prominent. The result of phytochemical test has been summarized in the Table 1.

**Table 1:** **Qualitative analysis of the phytochemicals of** ***Curcuma zedoaria***

Plant in extract	Tannins	Flavonoids	Saponins	Gums carbohydrate	Steroids	Alkaloids	Reducing sugar	Terpenoids
**Ethanolic rhizome extract of** ***Curcuma zedoaria***	+++	++	+++	+++	+++	+++	+++	+++

Symbol (+++) indicates presence in high concentration, Symbol (++) indicates presence in moderate concentration, Symbol (+) indicates presence in trace concentration.

### Analgesic activity

#### Hot plate method

**Figure 1:**
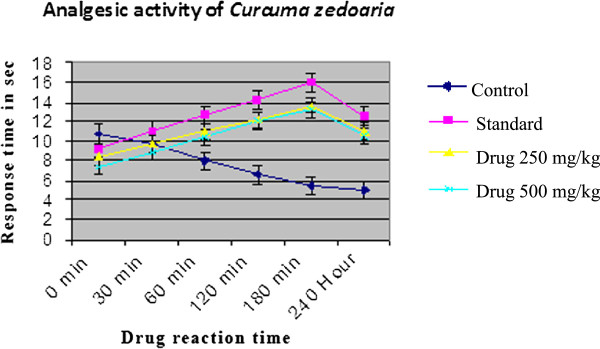
**Comparison graph of** ***Curcuma zedoaria*** **with control and standard.**

**Figure 2:**
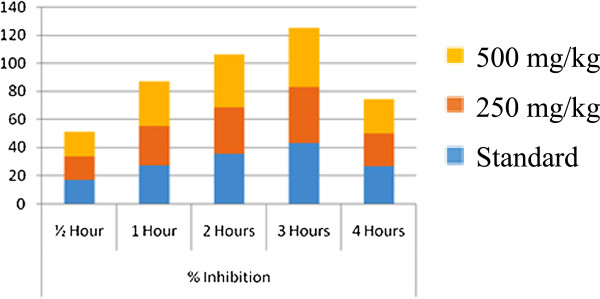
**Graph showing the % of inhibition of the ethanol extract of** ***Curcuma zedoaria*** **using the hot plate method.**

Results of hotplate test are presented in Table 2 and Table 3 for the crude extract of *C. zedoaria.* The rhizome extract of the plant significantly increased the reaction time of heat sensation in mice at the doses of 250 and 500 mg/kg BW and the percentage protection is almost equivalent to the respective doses (Figure 1). In the 3rd hour of study, the extract increased the reaction time of heat sensation to 61.99% and 78.22% at the doses of 250 and 500 mg/kg BW respectively while that of the standard drug was 74% and the results were found to be highly statistically significant (P < 0.001). The extract exhibited a dose dependent increase in latency time when compared with control (Figure 2).

**Table 2:** **Analgesic activity study of the ethanol extract of** ***Curcuma zedoaria*** **using the hot plate method**

Treatment	0 min	30 min	60 min	120 min	180 min	240 min
**Control**	10.70 ± 0.84676	9.6600 ± 0.93680	8.0000 ± 0.81425	6.5800 ± 0.64062	5.5200 ± 0.54900	5.0000 ± 0.44272
**Standard**	9.1400 ± 0.52402	11.0200 ± 1.00170*	12.6000 ± 0.94499***	14.1600 ± 1.07638***	15.9600 ± 0.67646***	12.4800 ± 0.69814**
**Drug250 mg/kg**	8.4200 ± 0.34409	9.7400 ± 0.42261	10.9800 ± 0.37336*	12.1800 ± 0.15620***	13.6400 ± 0.22045***	11.2100 ± 0.025768**
**Drug500 mg/kg**	7.4400 ± 0.60795	8.9200 ± 0.48724	10.4000 ± 0.43012*	12.0000 ± 0.20494***	13.2600 ± 0.14697***	10.0000 ± 0.24819**

All values are Mean ± SEM, n = 5. One way Analysis of Variance (ANOVA) followed by Dennett’s test was performed as the test of significance. The minimum value of p < 0.05 was considered significant. **p < 0.01, ***p < 0.001 as compared with control group.

**Table 3:** **Percentage inhibition of** ***Curcuma zedoaria*** **at different time intervals**

Treatment group	% inhibition				
	½ Hour	1 Hour	2 Hours	3 Hours	4 Hours
**Standard**	20.56	37.85	54.92	74.61	36.54
**250 mg/kg Extract**	15.67	30.40	44.65	61.99	33.13
**500 mg/kg extract**	19.89	39.78	53.20	78.22	34.4

#### Acetic acid-induced writhing test

**Figure 3:**
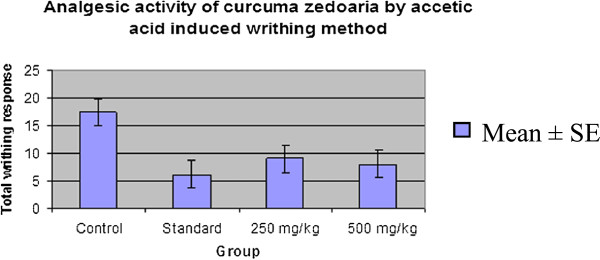
**Comparison curve of analgesic activity of** ***Curcuma zedoaria*** **by acetic acid induced writhing method with control and standard groups.**

**Figure 4:**
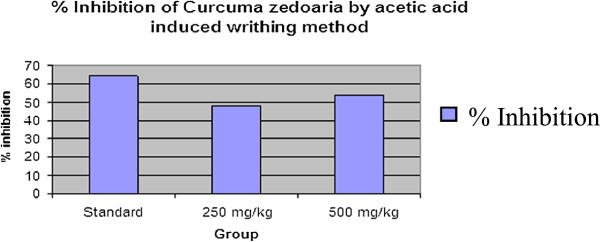
**Percentage inhibition of** ***Curcuma zedoaria*** **by acetic acid induced writhing method with standard.**

Inhibition of licking response in mice due to the administration of the test drugs during acetic acid-induced writhing test is shown in Table 4. The oral administration of both doses of *C. zedoaria* rhizome extract significantly (p < 0.001) attenuated the acetic acid-induced abdominal writhes in mice in a dose dependent fashion (Figure 3). The percent inhibition of writhing response by the extract was 48.28% and 54.02% at 250 and 500 mg/kg doses respectively while the standard Diclofenac sodium (10 mg/kg) showed 64.36% inhibition in comparison with the control (Figure 4).

**Table 4:** **Analgesic activity test of** ***Curcuma zedoaria*** **by Acetic Acid induced**

Treatment	Total Writhing Counts					Mean ± SE	% Inhibition
**Control**	19	11	16	15	26	17.4 ± 2.50	**-**
**Standard**	4	6	6	8	7	6.2 ± 0.66***	64.36
**250 mg/kg**	7	8	10	9	11	9 ± 0.71**	48.28
**500 mg/kg**	10	8	9	6	7	8 ± 0.71***	54.02

All values are Mean ± SEM, n = 5. One way Analysis of Variance (ANOVA) followed by Dennett’s test was performed as the test of significance. The minimum value of p < 0.05 was considered significant. *p < 0.05, **p < 0.01, ***p < 0.001 as compared with control group.

#### Formalin-induced writhing test

**Figure 5:**
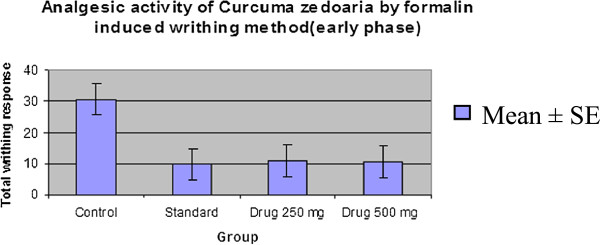
**Comparison curve of** ***Curcuma zedoaria*** **with control group and standard group (Early phase).**

**Figure 6:**
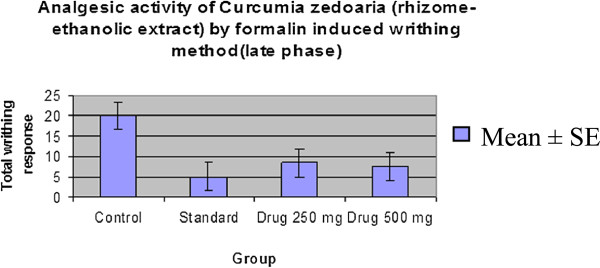
**Comparison curve of** ***Curcuma zedoaria*** **with control group and standard group (Late phase).**

**Figure 7:**
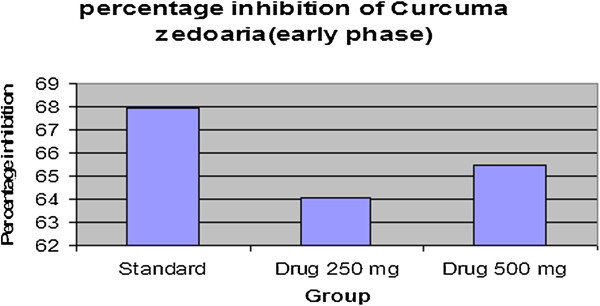
**Percentage inhibition graph of** ***Curcuma zedoaria*** **by formic acid (Early phase).**

**Figure 8:**
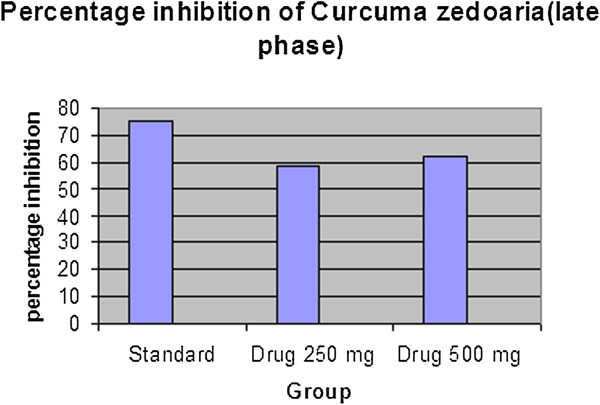
**Percentage inhibition graph of** ***Curcuma zedoaria*** **by formic acid (Late phase).**

The effect of the extract of *C. zedoaria* on formalin induced pain in mice is shown in Table 3. The extract significantly inhibited the licking response in both the early phase (64.04% at 250 mg/kg, p < 0.05 and 64.49% at 500 mg/kg, p < 0.01) and the late phase (58.42% at 250 mg/kg, p < 0.05 and 62.37% at 500 mg/kg, p < 0.01) of the formalin test which were comparable to those of the standard drug (Table 5 & Table 6, Figure 5, Figure 6, Figure 7 & Figure 8). Both these inhibitions were dose dependent.

**Table 5:** **Analgesic activity test of** ***Curcuma zedoaria*** **by formalin induced writhing method**

Early phase (0–5 min)		Late phase (20–30 min)	
**Group**	**Mean ± SE**	**Group**	**Mean ± SE**
Control	30.60 ± 5.3	Control	20.20 ± 4.86
Standard	9.8 ± 0.58***	Standard	5.0 ± 0.70***
Drug 250 mg	11.00 ± 0.71***	Drug 250 mg	8.40 ± 0.51**
Drug 500 mg	10.56 ± 0.58***	Drug 500 mg	7.60 ± 0.51***

All values are Mean ± SEM, n = 5. One way Analysis of Variance (ANOVA) followed by Dennett’s test was performed as the test of significance. The minimum value of p < 0.05 was considered significant. **p < 0.01, ***p < 0.001 as compared with control group.

**Table 6:** **Percentage inhibition of** ***Curcuma zedoaria*** **by formalin induced writhing method (Early phase and late phase)**

Early phase (0–5 min)		Late phase (20–30 min)	
**Treatment**	**% inhibition**	**Treatment**	**% inhibition**
Standard	67.97	Standard	75.25
*Drug* 250 mg	64.04	*Drug* 250 mg	58.42
*Drug* 500 mg	64.49	*Drug* 500 mg	62.37

### Anti-inflammatory test result

#### In vivo anti-inflammatory test result

**Figure 9:**
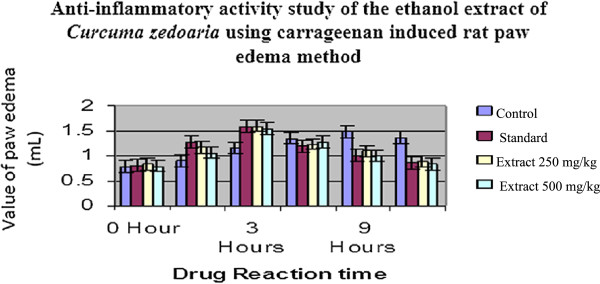
**Comparison chart of Anti-inflammatory of** ***Curcuma zedoaria*** **with Control & Standard group.**

**Figure 10:**
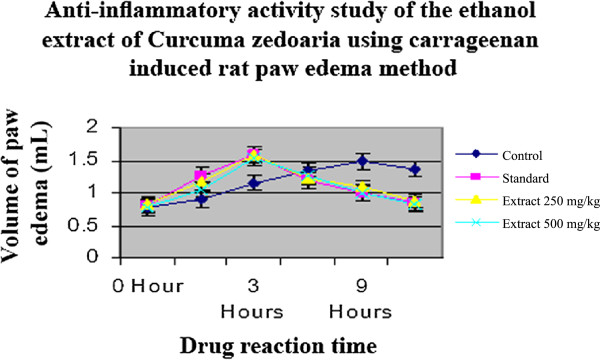
**Graph showing the anti-inflammatory activity of the ethanol extract of** ***Curcuma zedoaria*** **using carrageenan induced rat paw edema method.**

**Figure 11:**
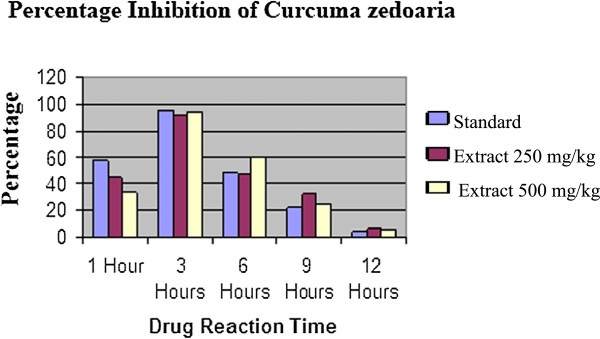
**Graph showing the % of inhibition of the ethanol extract of Curcuma zedoaria using carrageenan induced rat paw edema method.**

The anti-inflammatory effects of the extract and standard drug are presented in Table 4. In control animals, the subplantar injection of carrageenan produced a local edema that increased progressively to reach a maximal intensity 3 hours after injection (Figure 9 & Figure 10). The oral administration of both doses of the ethanolic rhizome extract of *C. zedoaria* significantly (p < 0.05, p < 0.01 and p < 0.001) inhibited inflammatory response induced by carrageenan in rats in a dose related manner (Table 7). The most prominent inhibition of 92.02% at 250 mg/kg and 94.67% at 500 mg/kg were observed at the 3rd hour of study after which the inhibitory activity was found to decline (Table 8 & Figure 11). The result was found to be highly statistically significant at 3rd hour after administration of the sample drugs (p < 0.001) (Table 7).

**Table 7:** **Anti-inflammatory activity study of the ethanol extract of** ***Curcuma zedoaria*** **using carrageenan induced rat paw edema method**

Treatment group	0 hour	1 hour	3 hours	6 hours	9 hours	12 hours
**Control**	0.7760 ± 0.02600	0.9040 ± 0.03855	1.1560 ± 0.04665	1.3520 ± 0.04247	1.4880 ± 0.03527	1.3700 ± .04159
**Standard**	0.8080 ± 0.01772	1.2720 ± 0.04521***	1.5820 ± 0.02417***	1.2020 ± 0.03541**	0.9900 ± 0.02608	0.8460 ± .00812
***C.zedoaria*** **250 mg/kg (n = 5)**	0.8280 ± .01772	1.1700 ± 0.07530**	1.590 ± 0.03619***	1.2240 ± 0.03043**	1.0940 ± 0.02909	0.8840 ± .02926
***C.zedoaria*** **500 mg/kg (n = 5)**	0.7920 ± .03153	1.0580 ± .03541**	1.5420 ± .03441***	1.2660 ± .04069**	0.9940 ± .02638	0.8320 ± .02396

All values are Mean ± SEM, n = 5. One way Analysis of Variance (ANOVA) followed by Dennett’s test was performed as the test of significance. The minimum value of p < 0.05 was considered significant. **p < 0.01, ***p < 0.001 as compared with control group.

**Table 8:** **Percentage inhibition of** ***Curcuma zedoaria*** **at different time intervals**

	% inhibition				
**Group**	**1 hour**	**3 hours**	**6 hours**	**9 hours**	**12 hours**
**Standard**	57.42	95.79	48.76	22.52	5.00
**250 mg/kg**	44.80	92.02	47.20	32.12	6.76
**500 mg/kg**	33.56	94.67	59.85	25.13	5.05

#### In vitro anti-inflammatory result

**Figure 12:**
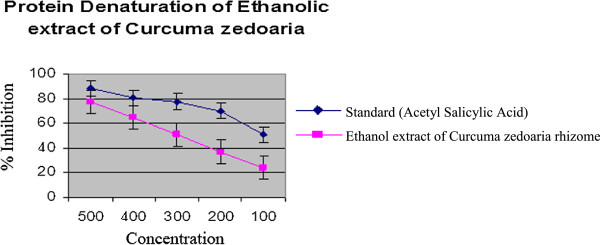
**Comparison curve of Acetyl Salicylic Acid with** ***Curcuma zedoaria*** **.**

In the present study for *in vitro* anti-inflammatory test, the ethanol extract, of *C.zedoaria* showed mean inhibition of protein denaturation of 77.15, 64.43, 53.04, 36.78 and 23.70% for doses of 500, 400, 300, 200 and 100 μg/mL respectively, whereas, for ASA it was found to be 88.06, 80.25, 77.18, 69.58 and 50.56% for same doses respectively (Table 9 & Figure 12). The ethanolic extracts of *C.zedoaria* showed good anti-inflammatory activity with a linear response. Maximum inhibition of 77.15 ± 2.93% was observed at 500 μg/mL and standard anti-inflammatory drug (Aspirin) showed the maximum inhibition, 88.06 ± 2.07% at the concentration of 500 μg/mL. The ability of extract of *C.zedoaria* to inhibit thermal denaturation of protein was found to be statistically significant (p < 0.05). In conclusion, further investigations are needed to identify the active constituents and the exact mechanism(s) of action responsible for the reported analgesic & anti-inflammatory properties of *Curcuma zedoaria*.

**Table 9:** **Protein denaturation activity of ethanol extracts of** ***Curcuma zedoaria***

Sample	Conc.	Mean% inhibition ± SD
	(μg/mL)	
Standard (Acetyl Salicylic Acid)	500	88.06 ± 2.07***
	400	80.25 ± 1.34***
	300	77.18 ± 1.45***
	200	69.58 ± 0.83***
	100	50.56 ± 1.36**
Ethanol Extract of *Curcuma zedoaria* (Rhizome)	500	77.15 ± 2.93***
	400	64.43 ± 4.27***
	300	53.04 ± 2.65**
	200	36.78 ± 5.16**
	100	23.70 ± 3.06*

All values are Mean ± SEM, n = 3. One way Analysis of Variance (ANOVA) followed by Dennett’s test was performed as the test of significance. The minimum value of p < 0.05 was considered significant. *p < 0.05, **p < 0.01, ***p < 0.001 as compared with control group.

## Discussion

Phytochemical investigation of the ethanolic rhizome extract of *Curcuma zedoaria* indicates the presence of tannins, alkaloids, flavonoids, gum and carbohydrates, reducing sugar and terpenoids. The presence of these chemicals represents the possibility of some biological activity of the ethanolic rhizome extract of *Curcuma zedoaria*.

A variety of pharmacological activities has been attributed to flavonoid compounds. Some flavonoids are reported to have analgesic and anti-inflammatory activities [21–23]. Some flavonoids can significantly inhibit a number of inflammatory mediators [24]. Terpenoids also possess significant analgesic and anti-inflammatory activities [25, 26]. Such activity has been attributed to the ability to inhibit phospholipase A2 and thereby ultimately blocking the metabolism of arachidonic acid [27]. A number of alkaloids may also prevent inflammation through blocking the metabolic pathway of arachidonic acid [28, 29].

The hot plate test measures the response to a brief, noxious stimulus having a close resemblance to clinical pain. This test measures the complex feedback to a non-inflammatory, acute nociceptive input and is one of the models normally used for studying central nociceptive activity [30]. The method is considered to be selective for the drugs acting centrally. It is a fact that any agent that causes a prolongation of the hot plate latency using this test must be acting centrally [31]. The ethanolic rhizome extract of *C. zedoaria* presented a longer latency time than the control group in the hot plate test in a dose related manner. Therefore, the extract has activity on central nervous system.

Acetic acid induced writhing response in mice, associated to visceral pain, finds much attention to evaluate peripherally active analgesics [32]. Pain sensation in acetic acid induced writhing method is obtained by generating localized inflammatory response resulting in release of free arachidonic acid from tissue phospholipid via cyclooxygenase and prostaglandin biosynthesis [33]. The boost in prostaglandin levels within the peritoneal cavity then raises inflammatory pain by rising capillary permeability [34]. The agent lessening the number of twitching will render analgesic effect preferably by restriction of prostaglandin synthesis, a peripheral mechanism of pain inhibition [33]. In the present study, the crude extract produced significant analgesic effect which might be due to the presence of analgesic principles acting with the prostaglandin alley.

The formalin test is a model of continuing pain including peripheral inflammation and central sensitization. The method exhibits a biphasic reaction comprising of an early (neurogenic) and a late (inflammatory) phase reaction and originates mainly from neurogenic inflammation followed by participation of kinins and leukocytes with their pro-inflammatory factors including prostaglandins [35]. It is also reported that acute inflammation convinced by formalin results from cell injury which serves the production of endogenous mediators [36]. Results of the present study show that the plant extract produced antinociception against both neurogenic and inflammatory phase of formalin induction. The fact that the extract at the doses tested produced analgesia in all nociceptive models is indicative that it possesses both central and peripheral antinociceptive effects and the mechanism of action of the extract could, in part, be related to lipooxygenase and/or cyclooxygenase of the arachidonic acid cascade and/or opioid receptors.

Carrageenan-induced inflammation is commonly used model for assessing the anti-inflammatory potency of compounds or natural products [37]. The probable mechanism of action of carrageenan-induced inflammation is bi-phasic, the first phase is characterised by the release of histamine, serotonin and kinins in the first hour; while the second phase is attributed to the release of prostaglandins and lysosome enzymes in 2 to 4 hours [38]. The second phase is sensitive to most clinically valid anti-inflammatory drugs [39]. The results of present study reveal that the extract significantly inhibited the carrageenan-induced acute inflammation in the 3^rd^hour of study and was comparable to that of the standard diclofenac sodium. So, the anti-inflammatory effect of *C. zedoaria* extract may be due to its suppressive action on prostaglandin, protease or lysosome synthesis or activity.

The method of anti-denaturation of egg albumin was chosen to evaluate anti-inflammatory property of *C.zedoaria*. In anti-denaturation assay the denaturation of egg albumin is induced by heat treatment. The denatured protein expresses antigens associated to Type III hyper-sensitive reaction which are related to diseases such as serum sickness, glomerulo-nephritis etc. [40]. Heat-denatured proteins are as effective as native proteins in provoking delayed hypersensitivity [41]. Moreover, it was already proved that conventional NSAID’s like phenylbutazone and indomethazine do not act only by the inhibition of endogenous prostaglandins production by blocking COX enzyme but also by prevention of denaturation of proteins [42]. Thus anti-denaturation assay is the convenient method to check the anti-inflammatory activity. From the result of the present study, the extract has shown considerable anti-inflammatory activity. *C.zedoaria* is capable of controlling the production of auto antigen and thereby it inhibits the denaturation of proteins and its effect was compared with the standard drug Aspirin. The secondary metabolites like phenolic compounds and tannins which were found in preliminary phytochemical screening might be responsible for this activity.

## Conclusion

Based on the present investigation, it can be concluded that the antinociceptive & in-vivo & in-vitro anti-inflammatory activity of the rhizome extract of *Curcuma zedoaria* might be attributed to the presence of the plant’s various secondary metabolites like tannins, saponins, steroids, alkaloids, reducing sugars, terpenoids and flavonoids. These experimental findings support the traditional use of this plant for the treatment of various ailments especially against pain and inflammatory conditions. However, further investigations are required to isolate the active constituents responsible for the observed effect, and to elucidate the possible mechanisms of action responsible for the antinociceptive and anti-inflammatory activities of the plant extract.
